# The Amygdala, Sleep Debt, Sleep Deprivation, and the Emotion of Anger: A Possible Connection?

**DOI:** 10.7759/cureus.2912

**Published:** 2018-07-02

**Authors:** Zahid Saghir, Javeria N Syeda, Adnan S Muhammad, Tareg H Balla Abdalla

**Affiliations:** 1 Department of Research, California Institute of Behavioral Neurosciences & Psychology, Fairfield, USA

**Keywords:** sleep, sleep deprivation, sleep anger, sleep frustration, sleep emotion, sleep mood

## Abstract

The association of sleep with emotions has long been studied. However, not much has been studied about the association of sleep deprivation and sleep debt with the emotion of anger. In this article, we focused on the association of sleep with anger. The results suggest that lack of sleep - sleep deprivation and sleep debt - both lead to the emotion of anger. Some hints about the involvement of the limbic system have been found. Yet, due to a lack of a quantity of evidence, we suggest that more studies are conducted on the same topic to help us have a more in-depth exploration of the association of lack of sleep with anger.

## Introduction and background

The effects of sleep on cognitive abilities and overall human health has been of interest in the scientific community for a long time. Studies have shown that sleep deprivation can affect human cognition and overall brain performance [1]. The effects of sleep deprivation are not just confined to brain function; in fact, it can affect other human organ systems as well [2-4]. There is a large body of research to support the connection between sleep, physical health, and neurological stability; there are fewer studies focusing on the impact of sleep deprivation on specific emotions such as anger. In this article, we will review current research that details the possible association of sleep with anger.

We conducted searches in PubMed, Google Scholar, Medline, and newspapers, and did a general Internet search to collect articles for this review. The keywords for the search included but were not limited to sleep, sleep deprivation, sleep anger, sleep frustration, sleep emotion, and sleep mood. Search results were limited to articles published within the last five years and which contained the keywords sleep deprivation and anger. Any studies involving animals were excluded. The inclusion-exclusion decision was made by the authors through mutual agreement. A total of 63,219 articles contained relevant information per the search criteria. Seventeen of the most relevant and comprehensive articles were selected for the purposes of this review.

## Review

Sleep and anger - a body of research evidence

Sleep is an essential part of our lives. The typical person needs seven to eight hours of sleep each night to maintain peak mental and physical health. Less than seven to eight hours of sleep can be harmful to human health. Getting less than adequate sleep is known as sleep deprivation [5]. When an individual has multiple consecutive days of sleep deprivation, they enter “sleep debt,” which is a cumulative effect of insufficient sleep for any period of time [6]. Because research has indicated a connection between sleep deprivation/sleep debt and mental health, it could be hypothesized that sleep debt could correlate with anger—irritability, aggression, and short temper. The effect of sleep deprivation on mood has been well-documented. The changes in mood that have been linked to sleep deprivation include anxiety, depression, mood swings, etc. This review focuses solely on the connection between sleep deprivation and anger, although there is less published research in this area.

Bauducco et al. [7] conducted a cross-sectional study to test the correlation between sleep deficit in adolescents and emotional and behavioral issues. Their sample included 2,767 students between the ages of 12 and 16. Fifty-two percent of the students studied were male. The study revealed that students who reported less than the recommended total sleep time (TST) experienced what Baudoccu et al. referred to as “norm-breaking behavior,” as well as emotional changes, including anger, depression, and anxiety. They concluded their research with the recommendation of good sleep practices by reducing sleep barriers such as technology, stress, and worry.

Itlani et al. [8] conducted a nationwide survey of Japanese high-school juniors and seniors to study the prevalence of anger and impulsivity and its associated factors. The survey questioned students on their personal data, lifestyle, mental status, and feelings of anger and impulsivity. The researchers used logistic regression (all P values < .05) to analyze a total of 94,777 responses. The researchers found a positive correlation between anger and impulsivity and behaviors such as “consuming alcohol, smoking, skipping breakfast, shorter sleep duration, decreased positive feelings, increased depressive feelings, and using a mobile phone for long hours." The researchers concluded by saying that healthy lifestyle choices, getting enough sleep every night, and having good mental health all play a key role in preventing issues with anger and impulsivity. A similarly designed study with first-year medical students as the research subjects found that the best predictors of sleep difficulties were stress, anger, hypervigilance, hostility, anxiety, and interpersonal sensitivity [9]. Studies such as these suggest a correlation between sleep deprivation and anger. All of the above-mentioned three studies more or less show similar results related to sleep and anger/aggression. However, there are currently no studies that have concluded a pathophysiological association between sleep and anger in adolescents.

Sleep deprivation appears to impact adults, adolescents, and children in similar ways. Sleep deprivation can exacerbate pre-existing mood disturbances, such as anger, depression, and anxiety, and can lead to confusion, fatigue, and lack of vigor. Even just one sleepless night correlates with these changes in function [5]. Randler et al. [10] administered the Buss-Perry Aggression Questionnaire, which assesses physical and verbal aggression, anger, and hostility in a group of young adult males and analyzed responses in correlation with sleep duration. They found that young males who reported shorter sleep durations had higher instances of aggression and anger. Researchers have noticed similar patterns of sleep deprivation and changes in mood and behavior in both male and female subjects. On average, males tend to score higher in physical and verbal aggression when sleep deprived [10] and females tend to be more susceptible to decreased mood, anxiety, low energy, and brain fog [5,11]. Male and female children score similarly when tested for the correlation between sleep deprivation and mood/behavior changes. However, these behavior/mood changes are more likely to present as externalizing behaviors, such as hyperactivity, anger, aggression, impulsivity, tantrum behavior, and inappropriate social interactions behaviors [12].

The amygdala is most commonly associated with its primary function as the emotional center of the brain. The amygdala additionally plays an important role in the mechanisms of sleep. When an individual is sleep deprived, a functional deficit occurs between the amygdala and the ventral anterior cingulate cortex (vACC), which can result in decreased mood and can cause the amygdala to have heightened responses to negative stimuli [13]. Sleep debt reduces the ability of the medial prefrontal cortex (MPFC) to suppress activity in the amygdala, leading to emotional instability [14]. A prolonged deprivation of rapid eye movement (REM) sleep is associated with functional changes in multiple brain regions [14] and can result in altered receptor activity, which can lead to mood alterations such as anger [15].

Motomura et al. [16] hypothesized that “resolution of Potential Sleep Debt (PSD) through sleep extension impacts mood by changing the functional connectivity between the prefrontal cortex and amygdala.” In their study, 15 male subjects were selected for a nine-day sleep extension followed by one night of total sleep deprivation (TSD). Following this 10-day intervention, they used magnetic resonance imaging (MRI) to evaluate regional cerebral blood flow (rCBF) in conjunction with a questionnaire on negative moods to assess the results of their study. They found that negative mood and amygdala rCBF were greatly decreased following a sleep extension. They further state in their article, “The amygdala had a significant negative functional connectivity with the medial prefrontal cortex (FCamg-MPFC), and this negative connectivity was greater after sleep extension than at BL." After TSD, these indices reverted to the same level as at baseline (BL). An additional path analysis with structural equation modeling showed that the FCamg-MPFC significantly explained the amygdala rCBF and that the amygdala rCBF significantly explained the negative mood. These findings suggest that the use of our sleep extension protocol normalized amygdala activity via negative amygdala-MPFC functional connectivity. The resolution of unnoticed PSD may improve mood by enhancing frontal suppression of hyperactivity in the amygdala caused by PSD accumulating in everyday life” [16]. If we look at the above-mentioned studies and analyze them, we will see that almost all of the above-mentioned studies unanimously agree on one point, that lack of sleep does impact the human emotional state. Moreover, the association of sleep with the amygdala, and with the amygdala being the part of the brain related with emotions, points towards the notion that sleep and emotions such as anger and aggression are associated with one another.

Moreover, in recent times, clinical hypnosis has been researched as a modality to cope with chronic headaches, anxiety symptoms, recurrent abdominal pain, depression, grief and bereavement, phobias, anger, family stressors, sleep disorders, or enuresis. This suggests a possible association of anger with sleep. It is quite obvious that when hypnosis, which is itself a mode of “sleep,” can help cope up with anger, there is a link between sleep deprivation and emotions such as anger. It is also suggestive of our idea that completing a full seven to eight hours of sleep can reduce the emotional symptoms of anger [17]. However, to comment on this with confidence, we need consistent future interventional studies for a longer period of time. Until then, we have to rely solely upon the available data.

## Conclusions

In this review article, we addressed the association of sleep with anger. Not many studies published in the last five years point towards this association. There is enough literature available on sleep and behavior; however, the association of sleep with anger or aggression is not readily available. There is a perceived connection between emotions and sleep due to the dual role the amygdala plays in both. A large body of research supports the connection between sleep deprivation and mood changes such as increased anger and aggression. Individuals who get an adequate amount of sleep each night exhibit fewer emotional outbursts, such as anger, and display fewer aggressive behaviors. These results are seen with minor differences between males and females and across various age groups. Prolonged sleep deprivation has been connected to changes in the brain such as reduced receptor sensitivity and changes in functional communication between brain regions. More research needs to be done to continue to evaluate the effects of sleep deprivation on mood and behavior (especially anger), particularly to highlight key differences in males and females and in children and adults. The consensus seems to be that getting an adequate amount of sleep each night promotes improved mood and health.
